# Commentary: Novel mtDNA methylation-associated prognostic signatures in colorectal cancer

**DOI:** 10.3389/fonc.2026.1780394

**Published:** 2026-02-19

**Authors:** Xin Jiang, Xu Zhang, Maonan Wang

**Affiliations:** 1 Changchun University of Chinese Medicine, Changchun, China; 2 Jilin Cancer Hospital, Changchun, Jilin, China

**Keywords:** colorectal cancer prognosis, metabolic-immune model, mitochondrial DNA methylation, regorafenib sensitivity, three-gene signature, tumor microenvironment

## 1 Introduction: challenges and new dimensions in precision therapy for colorectal cancer

Colorectal cancer (CRC) remains a leading cause of cancer-related mortality worldwide, presenting persistent challenges in clinical management. While immune checkpoint inhibitors (ICIs) have significantly improved outcomes for the approximately 15% of patients with microsatellite instability-high (MSI-H) tumors, the clinical benefit remains elusive for the vast majority (85%) of patients who present with microsatellite stable (MSS) or proficient mismatch repair (pMMR) “cold tumors”. A primary bottleneck in the treatment of these patients is the acute lack of reliable biomarkers to guide adjuvant therapy or the selection of targeted agents in advanced stages (1).

In this context, the study by Wang et al. published in Frontiers in Oncology, offers a significant paradigm shift by focusing on mitochondrial DNA (mtDNA) methylation—a burgeoning yet complex frontier in cancer epigenetics (2). Rather than relying on traditional nuclear genomic markers, the authors distill mitochondrial metabolic signals into a concise three-gene prognostic signature comprising TINAG, EPHB2, and FCN3. By linking these mitochondrial-derived signals to immune infiltration and therapeutic vulnerability, the study directly addresses the unmet clinical need for precise risk stratification and targeted intervention in the MSS/pMMR CRC population. This commentary evaluates the scientific rigor and the translational promise of this metabolic-immune framework in light of current bottlenecks in CRC precision oncology.

## 2 Core scientific debate: the “illusion” and “reality” of mitochondrial epigenetics

The study by Wang et al. is built upon the foundational theory that mtDNA methylation regulates tumor metabolism. However, it is important to note that the existence and functionality of mtDNA methylation have long been debated in the field of epigenetics. Due to the unique superhelical structure of the mitochondrial genome and the absence of histone protection, traditional bisulfite sequencing technology is prone to generating high levels of false-positive signals, a fact repeatedly confirmed in previous studies (3, 4).

To circumvent the technical pitfalls of direct detection, Wang et al. cleverly screened for “mtDNA methylation-associated genes” (MDM-RGs) encoded by the nuclear genome, rather than directly measuring mtDNA methylation levels. While this approach is feasible in bioinformatics analyses, it introduces the risk of ‘correlation, not causation.’ The model proposed in the study reflects disruptions to the nuclear genome transcriptome caused by mitochondrial dysfunction, rather than providing a direct mitochondrial epigenetic map. This highlights a key direction for future research: the use of advanced technologies such as Nanopore Sequencing or enzyme-based methylation sequencing (EM-seq) to rigorously verify the mtDNA methylation landscape in colorectal cancer (CRC). Nanopore sequencing offers a distinct advantage by enabling direct, single-molecule detection of methylated bases from native, long-read DNA without the need for bisulfite conversion (5). This bypasses false-positive signals that can arise from the superhelical structure of mtDNA and incomplete chemical conversion. Alternatively, EM-seq provides a non-destructive enzymatic approach that preserves DNA integrity, ensuring higher sensitivity and accuracy for mapping modifications within the compact mitochondrial genome compared to harsh bisulfite treatments (3, 4). These technical refinements will be pivotal in confirming whether the identified MDM-RG expression changes are truly driven by specific mtDNA methylation patterns, thereby establishing the biological causality of this axis.

## 3 Molecular mechanism analysis: biological rationality of the three-gene model

Although the starting point of the study is metabolic epigenetics, the three core genes identified in the model—EPHB2, FCN3, and TINAG—accurately capture several classic biological features of CRC’s aggressive progression, demonstrating high biological plausibility.

Firstly, EPHB2 (Ephrin type-B receptor 2) is a key tumor suppressor gene responsible for maintaining the intestinal crypt architecture. Numerous studies have shown that the loss of EPHB2 expression is associated with tumor cell dedifferentiation, epithelial-mesenchymal transition (EMT), and distant metastasis (6). The model by Wang et al. demonstrates that low expression of EPHB2 correlates with high-risk (poor prognosis), which aligns perfectly with previous findings regarding the inhibitory role of EphB2 in tumor invasion.

Secondly, FCN3 (Ficolin-3), a recognition molecule in the complement lectin pathway, is often underexpressed in “cold tumors,” where its low expression signals a loss of innate immune surveillance. In these tumors, impaired activation of dendritic cells prevents the recruitment of T-cell infiltration (7). The study found that the high-risk group was associated with an immunosuppressive microenvironment, potentially reflecting an immune evasion mechanism mediated by FCN3.

Finally, TINAG (Tubulointerstitial Nephritis Antigen), a matrix protein, is often highly expressed in pathological extracellular matrix (ECM) remodeling, providing a physical scaffold for tumor invasion (**Table 1**).

**Table 1:** Biological functions and clinical significance of key genes in Wang's model.

Gene symbol	Full name	Consensus function in CRC	Association in wang's model	Potential molecular mechanism
EPHB2	Ephrin type-B receptor 2	Tumor Suppressor; Maintains intestinal crypt architecture, inhibits invasion. Often lost during carcinogenesis.	Low expression = High risk; Negatively correlated with prognosis as an MDM-RG.	Maintains mitochondrial metabolic patterns required for differentiation; loss leads to stemness/glycolytic phenotype associated with mtDNA methylation changes.
FCN3	Ficolin-3	Innate Immune Recognition; Component of lectin pathway. Downregulated in various tumors.	Low expression = High risk; Correlated with immune infiltration status.	Low expression leads to impaired clearance of necrotic cells/mtDNA release, suppressing anti-tumor immune response, forming "cold tumors."
TINAG	Tubulointerstitial Nephritis Antigen	ECM protein; studied mostly in kidney, function in CRC unclear.	High expression = High risk; Novel prognostic factor.	Participates in pathological ECM remodeling, providing a physical scaffold for tumor invasion.

While direct functional research on TINAG in colorectal cancer (CRC) remains limited, its classification as a basement membrane–associated glycoprotein suggests that it is highly likely to participate in tumor progression. The ECM is increasingly recognized not merely as a structural scaffold but as a dynamic regulator of tumor biology, modulating processes such as cell proliferation, adhesion, migration, and invasion through biochemical and biomechanical cues within the tumor microenvironment. In CRC, dysregulated ECM remodeling influences metastatic potential and clinical outcomes by altering matrix composition and stiffness, and by engaging cell surface receptors such as integrins to activate downstream signaling networks that drive malignancy and therapeutic resistance (8, 9).

Integrin-mediated signaling, through focal adhesion complexes and associated kinases such as focal adhesion kinase (FAK), orchestrates bidirectional communication between cancer cells and the ECM, thereby facilitating cytoskeletal reorganization, epithelial–mesenchymal transition (EMT), and enhanced motility across the basement membrane and stromal barriers. These interactions have been shown to contribute significantly to CRC progression and metastasis, emphasizing the functional importance of ECM-integrin crosstalk in solid tumors (10). Given TINAG’s association with matrix architecture and cell–matrix adhesion, its elevated expression in high-risk CRC phenotypes may reflect augmented ECM remodeling and perturbed integrin signaling that promote malignant behavior.

Collectively, these observations underscore the necessity of systematic investigations into TINAG’s specific molecular interactions within the ECM and with integrin complexes in CRC models. Elucidating how TINAG influences ECM organization and integrin-dependent mechanotransduction will be crucial to define its precise role in tumor progression, invasion, and metastasis, and may reveal novel avenues for targeting the tumor microenvironment in precision oncology.

## 4 Clinical translation paradox and opportunities: from sorafenib to regorafenib

The most clinically insightful yet cautious aspect of this study is its prediction of drug sensitivity. Wang et al.’s analysis indicates that high-risk patients are more sensitive to sorafenib (2). However, clinical reality reveals a significant discrepancy: the Phase IIb RESPECT study demonstrated that sorafenib combined with chemotherapy failed to improve progression-free survival (PFS) or overall survival (OS) in metastatic CRC (mCRC), and it is thus not a standard treatment (11).

To bridge this “prediction vs. reality” gap, it is essential to clarify the bioinformatic and pharmacological link between sorafenib and regorafenib. From a bioinformatic perspective, drug sensitivity models (such as those derived from the GDSC or CTRP databases) often yield highly correlated results for drugs with similar pharmacophores. Regorafenib is a fluorinated derivative of sorafenib; they share a core biaryl urea structure and an overlapping inhibitory profile against key kinases, including VEGFR1-3, PDGFR-β, and the RAF signaling pathway (CRAF/BRAF). Consequently, a signature predicting sensitivity to sorafenib often serves as a surrogate marker for regorafenib sensitivity in high-throughput transcriptomic screens.

However, the clinical divergence between these two agents—highlighted by regorafenib’s success in the Phase III CORRECT study —stems from subtle but critical differences in their target spectrum and potency (12). Unlike sorafenib, regorafenib exerts more potent inhibition of VEGFR2, TIE2, and KIT. Furthermore, regorafenib notably influences the tumor microenvironment through CSF1R inhibition, which may modulate tumor-associated macrophages.

If the MDM-RG model truly reflects mitochondrial-driven metabolic reprogramming, its predictive value likely stems from the fact that mitochondrial dysfunction often exacerbates hypoxia and activates compensatory angiogenic pathways (VEGFR-dependent), rendering the tumor more vulnerable to broad-spectrum kinase inhibition. Therefore, future translational validation should not focus on the “predicted” sorafenib, but rather on retrospective analysis of regorafenib cohorts. Utilizing the MDM-RG model to identify patients who may derive the greatest benefit from regorafenib—particularly those who can tolerate its toxicity profile—would represent a major step toward precision metabolic-targeted therapy in advanced CRC.

## 5 Conclusion and prospects

In conclusion, the study by Wang et al. introduces a novel candidate biomarker for prognostic stratification in colorectal cancer by exploring the mitochondrial perspective. While the direct evidence for “mtDNA methylation” remains to be solidified, the three-gene model successfully integrates key malignant phenotypes such as tumor dedifferentiation (EPHB2), immune evasion (FCN3), and ECM remodeling (TINAG).

To address the challenges in this field, future research should focus on the following three areas: 1) using single-molecule sequencing technologies to directly map mtDNA methylation patterns in CRC, thereby confirming the mechanisms; 2) validating the predictive efficacy of the model in regorafenib-treated patient cohorts, filling the gap for biomarkers in advanced CRC precision therapy; 3) further exploring the role of mitochondrial dysfunction-induced cGAS-STING pathway activation in shaping the CRC immune microenvironment. This study provides important insights into the metabolic-immune interaction network in CRC, warranting further translational exploration.
